# Blended (Combined Spinal and General) vs. General Anesthesia for Abdominal Hysterectomy: A Retrospective Study

**DOI:** 10.3390/jcm12144775

**Published:** 2023-07-19

**Authors:** Stefano Catarci, Bruno Antonio Zanfini, Emanuele Capone, Francesco Vassalli, Luciano Frassanito, Matteo Biancone, Mariangela Di Muro, Anna Fagotti, Francesco Fanfani, Giovanni Scambia, Gaetano Draisci

**Affiliations:** 1 Department of Scienze Dell’Emergenza, Anestesiologiche e Della Rianimazione, IRCCS Fondazione Policlinico A. Gemelli, 00168 Rome, Italy; brunoantonio.zanfini@policlinicogemelli.it (B.A.Z.); emanuele.capone@policlinicogemelli.it (E.C.); luciano.frassanito@policlinicogemelli.it (L.F.); matteo.biancone@policlinicogemelli.it (M.B.); mariangela.dimuro@policlinicogemelli.it (M.D.M.); gaetano.draisci@policlinicogemelli.it (G.D.); 2 Department of Critical Care and Perinatal Medicine, IRCCS Istituto Giannina Gaslini, 16147 Genoa, Italy; 3 Department of Scienze della Salute della Donna, del Bambino e di Sanità Pubblica, IRCCS Fondazione Policlinico A. Gemelli, 00168 Rome, Italy

**Keywords:** spinal, blended anesthesia, hysterectomy, acute postoperative pain, women

## Abstract

Background: Adequate pain management for abdominal hysterectomy is a key factor to decrease postoperative morbidity, hospital length of stay and chronic pain. General anesthesia is still the most widely used technique for abdominal hysterectomy. The aim of this study was to assess the efficacy and safety of blended anesthesia (spinal and general anesthesia) compared to balanced general anesthesia in patients undergoing hysterectomy with or without lymphadenectomy for ovarian, endometrial or cervical cancer or for fibromatosis. Methods: We retrospectively collected data from adult ASA 1 to 3 patients scheduled for laparoscopic or mini-laparotomic hysterectomy with or without lymphadenectomy for ovarian, endometrial or cervical cancer or for fibromatosis. Exclusion criteria were age below 18 years, ASA > 3, previous chronic use of analgesics, psychiatric disorders, laparotomic surgery with an incision above the belly button and surgery extended to the upper abdomen for the presence of cancer localizations (e.g., liver, spleen or diaphragm surgery). The cohort of patients was retrospectively divided into three groups according to the anesthetic management: general anesthesia and spinal with morphine and local anesthetic (Group 1), general anesthesia and spinal with morphine (Group 2) and general anesthesia without spinal (Group 3). Results: NRS was lower in the spinal anesthesia groups (Groups 1 and 2) than in the general anesthesia group (Group 3) for every time point but at 48 h. The addition of local anesthetics conferred a small but significant NRS decrease (*p* = 0.009). A higher percentage of patients in Group 3 received intraoperative sufentanil (52.2 ± 18 mcg in Group 3 vs. Group 1 31.8 ± 16.2 mcg, Group 2 44.1 ± 15.6, *p* < 0.001) and additional techniques for postoperative pain control (11.4% in Group 3 vs. 2.1% in Group 1 and 0.8% in Group 2, *p* < 0.001). Intraoperative hypotension (MAP < 65 mmHg) lasting more than 5 min was more frequent in patients receiving spinal anesthesia, especially with local anesthetics (Group 1 25.8%, Group 2 14.6%, Group 3 11.6%, *p* < 0.001), with the resulting increased need for vasopressors. Recovery-room discharge criteria were met earlier in the spinal anesthesia groups than in the general anesthesia group (Group 1 102 ± 44 min, Group 2 91.9 ± 46.5 min, Group 3 126 ± 90.7 min, *p* < 0.05). No differences were noted in postoperative mobilization or duration of ileus. Conclusions: Intrathecal administration of morphine with or without local anesthetic as a component of blended anesthesia is effective in improving postoperative pain control following laparoscopic or mini-laparotomic hysterectomy, in reducing intraoperative opioid consumption, in decreasing postoperative rescue analgesics consumption and the need for any additional analgesic technique. We recommend managing postoperative pain with a strategy tailored to the patient’s physical status and the type of surgery, preventing and treating side effects of pain treatments.

## 1. Introduction

Adequate pain management for major gynecologic surgery is a key factor to decrease postoperative morbidity and increase patient satisfaction. Uncontrolled postoperative pain in this setting could result in a broad spectrum of harmful consequences, including increased morbidity, delayed hospital discharge and a higher incidence of persistent chronic pain [1,2].

Indeed, pain can modify a patient’s endocrine response by increasing catecholamines and cortisol levels and may amplify autonomic reflexes, triggering hypertensive crises or vagal syndromes which may result in severe complications during and after surgery. Furthermore, high levels of postoperative pain can negatively influence many “soft” outcomes, including limitation of physical functions, sleep quality and psychological status [3,4,5,6].

Despite the introduction of several guidelines, postoperative pain continues to be inadequately treated in gynecologic patients and poor pain control may reduce mobilization and significantly correlates with lower long-term quality of life and prolongation of hospital stay [2,7,8,9,10].

On the other hand, side effects of pain treatments (e.g., nausea and vomiting, paralytic ileus and delirium) are common in the immediate postoperative period: They can be severe and adversely affect patients’ postoperative recovery quality and patients’ satisfaction.

The ERAS guidelines represent a gold standard for the perioperative management of patients undergoing benign and oncological gynecological surgery based on the data obtained from the best clinical evidence available [2,7,8].

However, the optimal analgesic regimen for gynecological surgery with laparoscopy or mini-laparotomy is still the object of debate. Managing postoperative pain appropriately with a strategy tailored to the patient’s physical status and the specific gynecologic procedure, preventing and managing analgesia-related side effects, is crucial to optimize postoperative rehabilitation and to reduce hospital costs.

In literature, there has been increasing interest in the efficacy and safety of spinal anesthesia and intrathecal morphine as an adjunct to general anesthesia (blended) and for postoperative pain relief: Regional anesthesia is an effective way to increase pain control and minimize side effects of systemic opioid medications [11,12,13,14,15].

In obstetrics and gynecology, spinal anesthesia has become the gold standard for cesarean section and, in some patients, for vaginal hysterectomy. On the other hand, some previous studies have found no benefits from the neuraxial blockade, and general anesthesia is still the most widely used technique for abdominal hysterectomy [16].

The aim of this study was to assess the efficacy and safety of intrathecal administration of morphine with or without local anesthetic as a component of blended anesthesia compared to balanced general anesthesia in patients undergoing laparoscopic or mini-laparotomic hysterectomy with or without lymphadenectomy for ovarian, endometrial or cervical cancer or for fibromatosis.

## 2. Materials and Methods

### 2.1. Study Population and Data Collection

This observational retrospective study was approved by an independent Ethic Committee (Gemelli Ethic Committee ID 5421, protocol N 0040079/22). Written informed consent was obtained from each patient enrolled. This article adheres to the applicable STROBE guideline.

This study was conducted in the gynecological operating rooms and ward of the IRCCS Policlinico Agostino Gemelli Foundation of Rome, Italy; data were collected from 1 January 2019 to 31 December 2021.

Data were retrospectively collected from the acute pain service registry (no randomization was performed): All patients were followed by institutional Acute Pain Service and have been included in an electronic registry. 

We included adult ASA 1 to 3 patients scheduled for laparoscopic or mini-laparotomic hysterectomy (with an incision below the belly button), with or without lymphadenectomy for ovarian, endometrial or cervical cancer or for fibromatosis. Exclusion criteria were age below 18 years, ASA > 3, previous chronic use of analgesics, psychiatric disorders, laparotomic surgery with incision above the belly button and surgery extended to the upper abdomen for the presence of cancer localizations (e.g., liver, spleen or diaphragm surgery).

The cohort of patients was retrospectively divided into three groups according to the anesthetic management: general anesthesia and spinal with morphine and local anesthetic (Group 1), general anesthesia and spinal with morphine (Group 2) and general anesthesia without spinal (Group 3).

According to institutional protocols, in an outpatient ambulatory setting, patients were previously advised by an anesthesiologist that, as an adjunct to general anesthesia, they could receive spinal anesthesia before the induction of general anesthesia for postoperative pain management. 

On the day of surgery, the antalgic procedure was performed after the patient’s consent: Spinal anesthesia was performed in a sitting or lateral position, after appropriate skin disinfection with alcoholic 2% chlorhexidine and local anesthesia (2% lidocaine 3–5 mL), with a 25 G Whitacre needle at the L2-L3, L3-L4 or L4-L5 subarachnoid space, with equipotent doses of local anesthetics (0.5% isobaric levobupivacaine or 0.5% hyperbaric bupivacaine or 0.5% isobaric ropivacaine) and intrathecal opioid (morphine 50, 75 or 100 mcg) or with intrathecal opioid only (morphine 50, 75 or 100 mcg), depending on institutional protocol and the choice of the anesthesiologist in the operating room. The sensory level with pinprick was tested after 1 and 5 min in the supine position, in case of use of local anesthetic.

Afterwards, general anesthesia was induced with sufentanil 0.1–0.2 mcg/kg i.v., propofol 1.5–2 mg/kg i.v. and rocuronium bromide 0.6 mg/kg i.v. and subsequently maintained with inhalation anesthetic and opioids (sufentanil) keeping a MAC of 0.8–1.5 after endotracheal intubation. 

On the other hand, anesthesiologist performed only balanced general anesthesia according to the institutional protocol.

Dexamethasone 4 mg and ondansetron 4 or 8 mg were administered to all patients to reduce postoperative nausea and vomiting. 

Paracetamol 1 g four times daily and ketorolac 30 mg two times daily were prescribed postoperatively, if not contraindicated, in all the patients and additional medications (e.g., intravenous morphine managed via a Patient-Controlled Analgesia system) were administered as needed. Abdominal wall block (Transversus Abdominis Plane or Quadratus Lumborum block) or wound infiltration was also performed if spinal anesthesia had not been executed, according to the institutional protocol.

For each patient, age, weight, height, ASA (American Society of Anesthesiologists) classification, preoperative medications, type and duration of surgery, hemodynamic parameters during surgery, intraoperative drugs and analgesic technique were recorded. After the end of the surgery, patients were examined at regular intervals: after awakening before leaving the operating room, before the recovery room’s discharge and at 6, 12, 24 and 48 postoperative hours. 

Assessment of postoperative pain, using NRS (numerical rating scale), both at rest and with movement, was measured at each time (at the end of surgery, at the recovery room’s discharge and at 6, 12, 24 and 48 h) and the highest NRS was registered as the primary outcome. The regular assessment also included the following parameters: sedation (Ramsay Sedation Scale), PONV (postoperative nausea and vomiting), itching, motor block (Bromage classification), metallic taste in the mouth, speech disorders, visual disturbances, tinnitus and arrhythmias, further doses of analgesics as needed in addition to the hourly prescription of paracetamol and ketorolac, paralytic ileus and mobilization of patients in the postoperative period.

Additionally, the length of stay in the recovery room and hospital discharge were recorded for each patient.

### 2.2. Statistical Analysis

Data were reported as mean ± standard deviation for continuous variables or cases (percentage) for categorical variables. 

Continuous variables were tested for homogeneity of variances with Levene’s test and differences among groups evaluated by one-way ANOVA or Kruskal–Wallis as appropriate; pairwise comparisons among the means of two groups were performed with Bonferroni correction, and no pairwise comparison was needed for variables tested with Kruskal–Wallis. Categorical variables were analyzed with a chi-square test; pairwise comparisons among groups were made on a logistic regression model for the binary outcome, and Bonferroni correction was used. 

Repeated measurements in the first two postoperative days were evaluated with a linear mixed model for NRS scores (continuous) and a generalized linear mixed model for PONV and pruritus (categorical binary). Fixed factors included groups and time, and random factors included the patient. To account for differences in intraoperative opioids and rate of mini-laparotomy, the latter were also included as fixed factors in the models. A multivariate logistic regression was used to evaluate the impact of various factors on the occurrence of intraoperative hypotension.

Statistical significance was considered when the *p* value was <0.05. All analyses were performed with R Statistical Computing, version 4.1.

## 3. Results

A total of 644 patients undergoing hysterectomy were retrospectively included in the present analysis. They were further subdivided into three groups according to the anesthetic management: general anesthesia and intrathecal morphine and local anesthetic (Group 1, *n* 240), general anesthesia and intrathecal morphine (Group 2, *n* 255) and general anesthesia without spinal anesthesia (Group 3, *n* 149). Intrathecal morphine administration ranged from 50 to 100 mcg (100 mcg in more than 95% of patients), while intrathecal local anesthetic could be one of isobaric levobupivacaine (58.3%, 14 ± 1.7 mg), isobaric ropivacaine (23.8%, 14.6 ± 1.5 mg) and hyperbaric bupivacaine (15%, 13.7± 2.1 mg).

Baseline characteristics of the population and relevant surgical procedures are given in Table 1: No major differences were noted among the three groups, with the notable exception of mini-laparotomic incision occurring more frequently in Group 3 (Group 1 17.5%, Group 2 18.4%, Group 3 32.9%, *p* < 0.05). 

**Figure 1:**
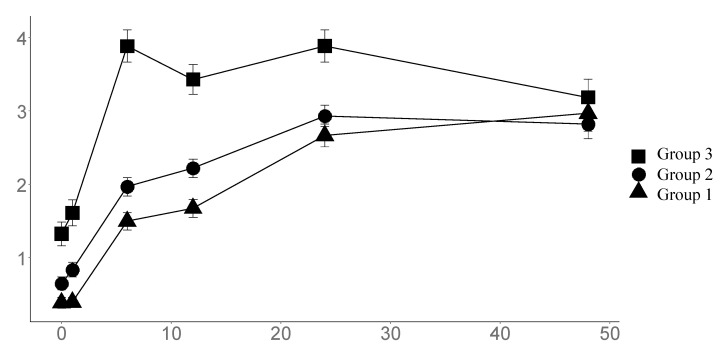
NRS registered at each visit.

The time course of postoperative pain scores is shown in Figure 1: The highest NRS measured at each visit was lower in the spinal anesthesia groups (Groups 1 and 2) than in the general anesthesia group for every time point; but at 48 h, they increased over time up to a plateau, and the increase was less steep for the spinal anesthesia groups (linear mixed model: time *p* < 0.001, groups *p* < 0.001, time:group interaction *p* < 0.001). The addition of local anesthetics conferred a small but significant NRS decrease (*p* < 0.05). 

Intraoperative management is also displayed in Table 2: While surgical time was similar (about 160 min), intraoperative intravenous sufentanil administration was lower when spinal anesthesia was performed, especially with the addition of local anesthetics (Group 1 31.8 ± 16.2 mcg, Group 2 44.1 ± 15.6, Group 3 52.2 ± 18 mcg) (*p* < 0.001).

Accordingly, 33.6% of Group 3 patients needed a rescue analgesic therapy in the first 24 h vs. 3.8% in Group 1 and 4.3% in Group 2. In a multivariate linear mixed model, including the amount of intraoperative sufentanil and the rate of mini-laparotomy as potential confounders, the time course of NRS scores was dependent only on the anesthetic technique (estimate −1.05, *p* < 0.001) and type of incision (estimate +0.35, *p* = 0.01).

A higher percentage of patients in Group 3 received additional techniques for postoperative pain control, including abdominal wall block or wound infiltration (11.4% in Group 3 vs. 2.1% in Group 1 and 0.8% in Group 2, *p* < 0.001), intravenous morphine administration (16% in Group 3 vs. 0% in Group 1 and 0.4% in Group 2, *p* < 0.001) and ketamine administration (12.8% in Group 3 vs. 0.4% in Group 1 and 2% in Group 2, *p* < 0.001). Also, more patients in Group 3 received either a PCA with morphine (43.6% in Group 3 vs. 1.3% in Group 1 and 0.4% in Group 2, *p* < 0.001) or an elastomeric pump with tramadol (15.4% in Group 3 vs. 0.4% in Group 1 and 0% in Group 2, *p* < 0.001).

**Figure 2:**
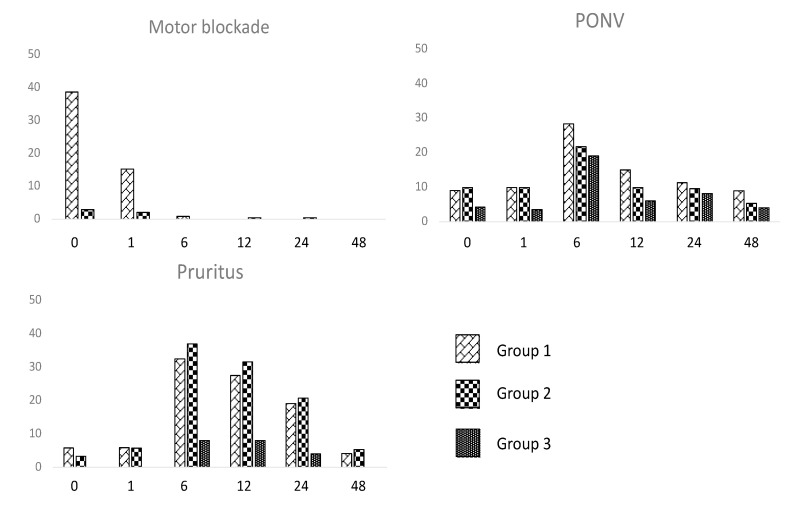
Percentage of patients with motor blockade, PONV and pruritus at each visit.

On the other hand, motor blockade (Bromage score > 1) was observed in up to 39% of Group 1 patients immediately after surgery but was negligible after 6 h (Figure 2). As shown in Figure 2, pruritus and PONV were present in up to one-third of patients: They both peaked at 6 h and decreased over time in all groups. Both pruritus and PONV were more frequent in the spinal anesthesia groups, but the incidence of pruritus was 3–4 times higher after intrathecal morphine (OR 10.1 in Group 1 and 12.6 in Group 2 vs. Group 3, *p* < 0.001), while PONV was only slightly more frequent in Group 1 (OR 3) and Group 2 (OR 2.3) than in Group 3 (*p* = 0.001).

Hypotension, defined as MAP (mean arterial pressure) < 65 mmHg lasting more than 5 min, was more frequent in patients receiving spinal anesthesia, especially with local anesthetics (Group 1 25.8%, Group 2 14.6%, Group 3 11.6%, *p* < 0.001), with the resulting increased need for vasopressors (norepinephrine, 5.8% in Group 1, 0.8% in Group 2, 2% in Group 3, *p* = 0.003) and inotropes (ephedrine, 17.1% in Group 1, 11.8% in Group 2, 6.71% in Group 3). On the contrary, fluid management was not significantly different among groups, with similar amounts of crystalloids, albumin and red blood cell transfusions. In addition, the urine output at the end of surgery was remarkably similar among the groups (Table 2). Factors associated with intraoperative hypotension in a multivariate logistic regression include the type of anesthesia (OR 2.7 for Group 1 *p* = 0.003, OR 1.3 for Group 2 *p* = 0.47) and age (OR 1.04 per year *p* = 0.002), but not BMI, baseline hypertension, ASA physical status and type of incision. Indeed, the mean age of patients with significant intraoperative hypotension was significantly greater (57.8 ± 11.1 vs. 52.9 ± 10.4, *p* < 0.001). 

Postanesthesia care unit (PACU) discharge criteria, including pain control, degree of motor blockade, PONV and pruritus, were met earlier in the spinal anesthesia groups than in the general anesthesia group (Group 1 102 ± 44 min, Group 2 91.9 ± 46.5 min, Group 3 126 ± 90.7 min, *p* < 0.05). No differences were noted in postoperative mobilization or duration of ileus. A higher percentage of patients receiving spinal anesthesia was discharged on postoperative day 2 (Group 1 43.4%, Group 2 43.7%, Group 3 31.9%, *p* < 0.05) (Table 3), but the association between early hospital discharge and spinal technique disappeared when accounting the type of incision as a confounder.

With regard to side effects related to the spinal, in this cohort of patients, we recorded one postdural puncture headache and one patient reporting a transient loss of sensibility at the level of the thigh which resolved after four weeks.

## 4. Discussion

Gynecological surgeries are some of the most frequent surgeries; however, 4.7–26.2% of women experience chronic postoperative pain [17]. Adequate postoperative pain management with a strategy tailored to the patient and surgery is crucial to improve the patient’s postoperative rehabilitation and reduce chronic postoperative pain. In gynecologic surgery, neuraxial block with opioids alone or combined with local anesthetics may be a proper way to block visceral nociceptive stimulus [11,14,15]. Indeed, neuraxial block utilizes anatomically targeted local anesthetics and adjuvants that interrupt afferent transmission and modulate the neuroendocrine and inflammatory response [18]. 

The primary finding of this large retrospective analysis was that subarachnoid morphine with or without local anesthetic was effective in improving postoperative pain control following laparoscopic or mini-laparotomic hysterectomy, in reducing intraoperative opioid consumption, in decreasing postoperative rescue analgesics consumption and the need for any additional analgesic technique.

Indeed, all groups in the study achieved acceptable pain control at each visit (NRS < 4): Patients who did not receive subarachnoid anesthesia needed a greater amount of intraoperative opioids, postoperative rescue analgesics and additional techniques were used to manage pain in the perioperative period. Nevertheless, pain scores in the subarachnoid anesthesia groups were always lower throughout the study period, even after accounting for major confounding factors (type of incision).

This result revealed that the spinal block was effective in reducing postoperative pain in our patients and allowed a significant opioid-sparing effect, with an expectable reduction in associated side effects. This outcome is consistent with previous findings in other surgeries: Levy BF et al. found that spinal anesthesia with intrathecal morphine may facilitate early mobilization in colorectal surgery [19]; similarly, Young J et al. found that intrathecal morphine ensures a reduction in postoperative opioid consumption in colorectal surgery [20].

Moreover, in accordance with previous studies [21,22,23,24,25,26], patients who received general anesthesia alone received several additional techniques to achieve sufficient postoperative pain control, including intraoperative opioids, abdominal wall blocks and wound infiltration.

The analgesic effect of subarachnoid administration of morphine lasts for about 24 h while the addition of local anesthetic produces an effect for about 6–12 h [18].

It is important to note that, while it is well known that neuraxial blocks have demonstrated efficacy regarding improved pain control and reduced postoperative opioid consumption in open surgery, the literature is still scarce in laparoscopic or mini-laparotomic surgery (with incision below the umbilicus) with expected postoperative pain from moderate to severe. However, these potential benefits must be weighed against the potential risks of neuraxial anesthesia, including possible delayed time to ambulation and voiding, and increased hospital length of stay [16].

Furthermore, it must be pointed out that the spinal technique is easier to perform and more manageable than peridural catheter placement; therefore, it seems to be appropriate for this type of surgery (laparoscopy or mini-laparotomy) as a component within a multimodal strategy.

Additionally, the motor block induced by the local anesthetic resulted in better pain control without interfering with patient’s mobilization in the ward. Adding local anesthetic to spinal block produced a relevant motor blockade in a significant group of patients (Bromage score > 1 in up to 39% of Group 1 patients) immediately after surgery, but the motor blockade was negligible after 6 h with no effects in postoperative mobilization. Also, no differences in postoperative ileus were registered.

On the other hand, it must be highlighted that adding local anesthetic to intrathecal morphine was associated with a benefit in pain control, at the price of increased intraoperative events and the need for vasopressor. Hypotensive events lasting more than 5 min (MAP < 65 mmHg) were significantly more frequent in patients receiving spinal block with local anesthetics with the resulting increased need for vasopressors and inotropes (norepinephrine and ephedrine). On the contrary, fluid management was not significantly different among groups, with similar amounts of crystalloids, albumin and red blood cell transfusions; also, the urine output at the end of surgery was similar among the groups. We conducted a multivariate logistic regression to establish factors associated with intraoperative hypotension: In our patients, the type of anesthesia and age, but no other features, were associated with hypotension. We recommend individualizing anesthetic strategy on the patient’s physical status and age to properly choose in which patient to administer spinal block with local anesthetics: It seems reasonable to avoid spinal block with local anesthetic in elderly, dehydrated patients or in patients at risk for hemodynamic instability.

Complications directly related to spinal procedure were rarely observed: The main side effects of subarachnoid analgesia were pruritus and PONV, while PDPH and neurological sequelae were uncommon. The incidence of pruritus was 3–4 times higher until 24 postoperative hours after administration of intrathecal morphine, while PONV was only slightly more frequent in patients receiving spinal, and no differences were registered after PACU discharge. These results are in accordance with other studies: The main disadvantages of intrathecal morphine include nausea, pruritus, urinary retention and doses greater than 0.2 mg may increase side effects without additional benefit [18]. 

Interestingly, earlier PACU discharge was possible when spinal anesthesia was administered with a significant reduction in time spent by the patient in the recovery room and the work of the staff who assisted her; moreover, a higher percentage of our patients receiving spinal block was discharged home on postoperative day 2, but in this case, we failed to find a significant independent association with the type of anesthesia (the significance disappears after stratifying patients also for the type of incision). Although this is not the purpose of the retrospective analysis, a reduction in personnel work in PACU and ward, and a reduction in hospital costs in treating our patients, is predictable.

This study has also important limitations due to the retrospective design, but the large cohort study suggests that spinal block with morphine alone or with local anesthetics may be a simple and very fast way to confer an advantage in pain control following laparoscopic or mini-laparotomic hysterectomy. Another substantial limitation of the study is the higher presence of patients undergoing mini-laparotomy in the general anesthesia group compared to the spinal groups; mini-laparotomy under the umbilicus was performed in some cases by the surgeon to extract the uterus at the end of the surgery. The visceral manipulation during surgery was similar in all the groups and the duration did not differ among the groups. In any case, the multivariate analysis considered this confounding factor, and its effect on pain scores was overall low but still significant. 

Randomized controlled trials should be developed to better define the most effective combinations and doses of intrathecal analgesics in laparoscopic and mini-laparotomic hysterectomy.

## 5. Conclusions

This large retrospective study shows that intrathecal administration of morphine with or without local anesthetic as a component of blended anesthesia is effective in improving postoperative pain control following laparoscopic or mini-laparotomic hysterectomy, reducing intraoperative opioid consumption, decreasing postoperative rescue analgesics consumption and the need for any additional analgesic technique. We recommend managing postoperative pain with a strategy tailored to the patient’s physical status and the type of surgery, preventing and treating analgesia-related side effects.

## Figures and Tables

**Table 1:** Preoperative characteristics and surgical procedure.

	Spinal (LA + Morphine) (*n* = 240)	Spinal (Morphine) (*n* = 255)	General (*n* = 149)	*p* Value
Age (years)	54 ± 11	54 ± 11	54 ± 10	ns
Weight (kg)	66 ± 13	66 ± 12	65 ± 12	ns
BMI (kg∙m^−2^)	24.8 ± 4.6	24.5 ± 4.7	24.5 ± 4.3	ns
Hypertension (*n*, %)	41 (17.1)	40 (15.7)	26 (17.4)	ns
Diabetes mellitus (*n*, %)	7 (2.92)	3 (1.18)	7 (4.7)	ns
ASA status				ns
ASA 1 (*n*, %)	14 (10.9)	34 (14.8)	27 (12.9)	
ASA 2 (*n*, %)	105 (82)	186 (80.9)	177 (84.7)	
ASA 3 (*n*, %)	9 (7)	10 (4.3)	5 (2.4)	
Diagnosis				ns
Ovarian cancer (*n*, %)	24 (16.1)	50 (19.6)	48 (20)	
Endometrial cancer (*n*, %)	56 (37.6)	95 (37.3)	89 (37.1)	
Cervical cancer (*n*, %)	17 (11.4)	25 (9.8)	17 (7.1)	
Fibromatosis (*n*, %)	35 (23.5)	65 (25.5)	71 (29.6)	
Other (*n*, %)	17 (7)	20 (7.8)	15 (10)	
Surgical technique				
Lymphadenectomy (*n*, %)	46 (19.2)	47 (18.4)	20 (13.4)	ns
Omentectomy (*n*, %)	18 (7.5)	14 (5.5)	6 (4)	ns
Mini-laparotomy (*n*, %)	42 (17.5)	47 (18.4)	49 (32.9) *$	<0.05

Data are expressed as mean ± standard deviation or count (percentage) as appropriate. *p* value refers to ANOVA for continuous variables and the chi-square test for categorical values. In the case of significant ANOVA and pairwise comparison, * indicates *p* value < 0.05 vs. Group 1 and $ *p* value < 0.05 vs. Group 2. ns = not statistically significant.

**Table 2:** Intraoperative management.

	Spinal (LA + Morphine) (*n* = 240)	Spinal (Morphine) (*n* = 255)	General (*n* = 149)	*p* Value
Surgery duration (min)	158 ± 55	163 ± 59	167 ± 68	ns
Intraoperative intravenous sufentanil (mcg)	32 ± 16	44 ± 16 *	52 ± 18 *$	<0.001
Intraoperative intravenous morphine (*n*, %)	0 (0)	1 (0.4)	24 (16.1) *$	<0.001
Intraoperative intravenous ketamine (*n*, %)	1 (0.4)	5 (2)	19 (12.8) *$	<0.001
Abdominal wall block or wound infiltration (*n*, %)	5 (2.1)	2 (0.8)	17 (11.4) *$	<0.001
PCA morphine (*n*, %)	3 (1.3)	1 (0.4)	65 (43.6) *$	<0.001
Elastomeric pump tramadol (*n*, %)	1 (0.4)	0 (0)	23 (15.4) *$	<0.001
Crystalloids (mL)	1647 ± 675	1592 ± 510	1609 ± 647	ns
Urine output (mL)	402 ± 314	359 ± 216	465 ± 667	ns
Albumin use (*n*, %)	10 (4.2)	6 (2.4)	4 (2.7)	ns
Blood transfusion (*n*, %)	5 (2.1)	3 (1.2)	4 (2.7)	ns
Hypotension (*n*, %)	61 (25.8)	35 (14.6)	17 (11.6)	<0.001
Ethylephrine use (*n*, %)	74 (30.8)	62 (24.3)	31 (20.8)	ns
Ephedrine use (*n*, %)	41 (17.1)	30 (11.8)	10 (6.7) *	ns
Atropine use (*n*, %)	16 (6.7)	13 (5.1)	4 (2.7)	ns
Norepinephrine use (*n*, %)	14 (5.8)	2 (0.8 *)	3 (2.01)	<0.05

Data are expressed as mean ± standard deviation or count (percentage) as appropriate. *p* value refers to ANOVA for continuous variables (Kruskal–Wallis for crystalloids and urine output) and chi-square test for categorical values. In case of significant ANOVA and pairwise comparison, * indicates *p* value < 0.05 vs. Group 1 and $ *p* value < 0.05 vs. Group 2. ns = not statistically significant.

**Table 3:** Postoperative outcomes.

	Spinal (LA + Morphine) (*n* = 240)	Spinal (Morphine) (*n* = 255)	General (*n* = 149)	*p* Value
Recovery room, time (min)	102 ± 44	92 ± 47	126 ± 91 $	<0.05
Postoperative mobilization (day)	1.3 ± 0.5	1.3 ± 0.5	1.3 ± 0.5	ns
Postoperative ileus (day)	1.3 ± 0.5	1.4 ± 0.6	1.4 ± 0.5	ns
Postdural puncture headache (*n*, %)	0 (0)	1 (0.8)	0 (0)	ns
Rescue analgesic therapy during the first 24 h (*n*, %)	9 (3.8)	11 (4.3)	50 (33.6 *$)	<0.001
Discharge on postoperative day 1 (*n*, %)	8 (3.4)	2 (0.8)	2 (1.34)	ns
Discharge on postoperative day 2 (*n*, %)	96 (43.4)	104 (43.7)	46 (31.9)	<0.05

Data are expressed as mean ± standard deviation or count (percentage) as appropriate. *p* value refers to ANOVA for continuous variables and chi-square test for categorical values. In case of significant ANOVA and pairwise comparison, * indicates *p* value < 0.05 vs. Group 1 and $ *p* value 0.05 vs. Group 2. ns = not statistically significant.

## Data Availability

Data are unavailable due to privacy or ethical restrictions.
